# Single-cell RNA sequencing reveals developmental heterogeneity among *Plasmodium berghei* sporozoites

**DOI:** 10.1038/s41598-021-82914-w

**Published:** 2021-02-22

**Authors:** Anthony A. Ruberto, Caitlin Bourke, Nicolas Merienne, Thomas Obadia, Rogerio Amino, Ivo Mueller

**Affiliations:** 1 Department of Parasites and Insect Vectors, Institut Pasteur, Paris, France; 2 Division of Population Health and Immunity, Walter and Eliza Hall Institute of Medical Research, Parkville, VIC Australia; 3 Department of Medical Biology, University of Melbourne, Melbourne, VIC Australia; 4 Hub de Bioinformatique et Biostatistique – Département Biologie Computationnelle, Institut Pasteur, 75015 Paris, France

**Keywords:** Transcriptomics, Malaria, Parasitology

## Abstract

In the malaria-causing parasite’s life cycle, *Plasmodium* sporozoites must travel from the midgut of a mosquito to the salivary glands before they can infect a mammalian host. However, only a fraction of sporozoites complete the journey. Since salivary gland invasion is required for transmission of sporozoites, insights at the molecular level can contribute to strategies for malaria prevention. Recent advances in single-cell RNA sequencing provide an opportunity to assess sporozoite heterogeneity at a resolution unattainable by bulk RNA sequencing methods. In this study, we use a droplet-based single-cell RNA sequencing workflow to analyze the transcriptomes of over 8000 *Plasmodium berghei* sporozoites derived from the midguts and salivary glands of *Anopheles stephensi* mosquitoes. The detection of known marker genes confirms the successful capture and sequencing of samples composed of a mixed population of sporozoites. Using data integration, clustering, and trajectory analyses, we reveal differences in gene expression profiles of individual sporozoites, and identify both annotated and unannotated markers associated with sporozoite development. Our work highlights the utility of a high-throughput workflow for the transcriptomic profiling of *Plasmodium* sporozoites, and provides new insights into gene usage during the parasite’s development in the mosquito.

## Introduction

Malaria is a burden on global public health with 228 million cases and 405,000 deaths estimated in 2018^1^. The disease is the result of an infection by a *Plasmodium* parasite, transmitted via the bite of a female *Anopheles* mosquito. In humans, *P. falciparum* and *P. vivax* cause most cases of the disease^1^. Despite progress in reducing the global malaria burden, the parasite still poses a major threat to millions of lives^2,3^.

An attractive target for intervention measures is the parasite in the sporozoite stage of its life cycle. Before it can infect a mammalian host, a *Plasmodium* sporozoite must travel from the midgut (MID) of a mosquito to the salivary gland (SG). Only a fraction of sporozoites, however, complete the journey^4^. Since SG invasion is required for transmission of sporozoites to a vertebrate host, insights at the molecular level may help contribute to strategies for malaria prevention. Extensive work has been performed in the phenotypic profiling of sporozoites, both as they develop in the mosquito and in their journey to the liver^5–7^. Furthermore, a number of genome-wide analyses generated from various *Plasmodium* species using bulk RNA sequencing (RNA-seq) methods have elucidated important transcriptional profiles of sporozoites^8–13^.

Recent advances in single-cell RNA-seq (scRNA-seq) methods have prompted new ways of deriving biological insights unattainable by bulk RNA-seq efforts^14,15^. However, the use of scRNA-seq to explore gene expression patterns across the different *Plasmodium* parasites is still a nascent area of research^16–23^. Only two studies^17,20^ have previously assessed the transcriptional profiles of MID and SG sporozoites at single-cell resolution.

scRNA-seq requires partitioning and lysis of single cells, conversion of RNA into cDNA, and cDNA amplification to generate sequencing libraries. There are various methods available to accomplish these tasks, each of which have their own advantages and disadvantages^24,25^. To date, only the plate-based Smart-seq2 technology^26^ has been used to generate *Plasmodium* sporozoite scRNA-seq data^17,20^. Despite its high gene coverage, the plate-based nature of the protocol makes detection of rare cell populations difficult due to the considerably fewer cells that can be simultaneously processed. Complementary approaches to low-throughput, plate-based protocols are high-throughput droplet-based methods^27–29^. At the cost of coverage, the ability of these methods to capture hundreds to thousands of cells in a single experiment makes it possible to detect rare cell populations.

The goal of the present study was twofold: first, to test whether a high-throughput droplet-based single-cell capture platform (10x Genomics) could be used to profile sporozoites at single-cell resolution; and second, to design a workflow to analyze transcriptomes obtained from thousands of individual sporozoites in order to expand the limited body of knowledge on sporozoite biology at single-cell resolution.

In what follows, we show that 10x Genomics’ droplet-based single-cell technology is an effective, high-throughput method for partitioning sporozoites and generating scRNA-seq data. We profile the transcriptomes of over 8000 *P. berghei* ANKA sporozoites derived from SGs and MIDs of mosquitoes 21 days after an infectious blood meal. Then, we identify clusters of sporozoites with varying gene expression profiles that suggest different developmental states among sporozoites. Last, we use these clusters as inputs for trajectory and gene enrichment analyses, and offer novel insights into sporozoite biology at single-cell resolution.

## Results

### Strategy to capture sporozoites and measure gene expression at single-cell resolution

**Figure 1:**
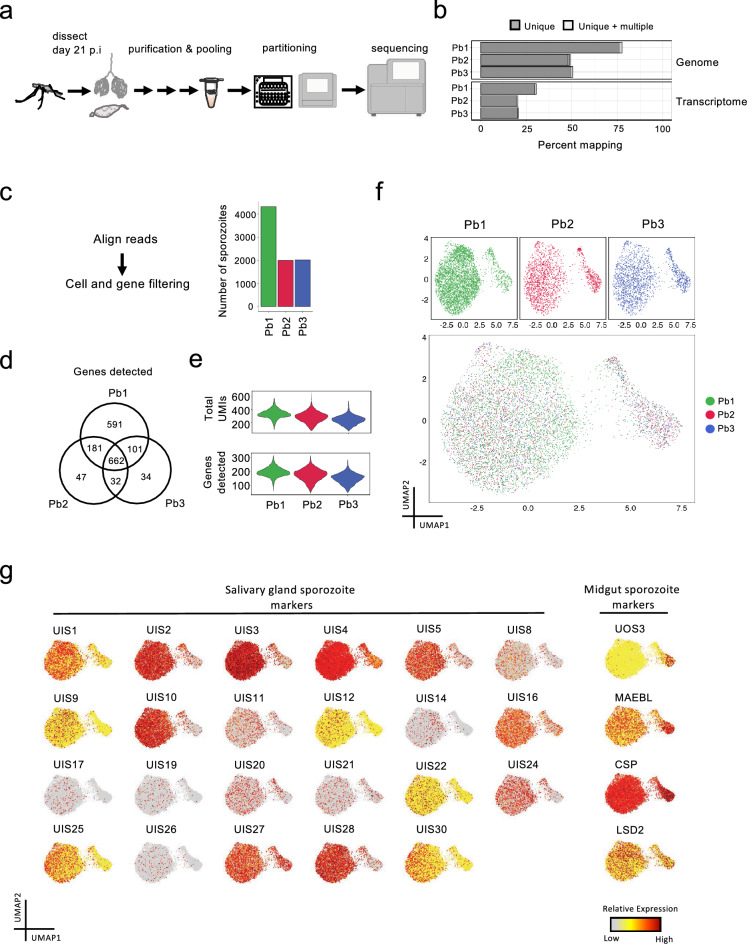
Mapping, quality control, and integration of *P. berghei* sporozoite 10 × scRNA-seq data. (**a**) Schematic illustrating the workflow used for scRNA sequencing of *P. berghei* sporozoites. (**b**) Number of *P. berghei* reads mapping to the genome (top) and transcriptome (bottom) across the three libraries. (**c**) Number of sporozoite transcriptomes analyzed post cell and gene filtering. (**d**) Number of unique and overlapping transcripts across the three single-cell library preparations. (**e**) Violin plots showing the distribution of total UMIs (top) and unique transcripts (bottom) across each of the three replicates. (**f**) UMAP representations displaying integrated scRNA-seq sporozoite datasets individually (top panels) and overlaid (bottom panel). (**g**) UMAP representations displaying the expression of UIS markers (left) and MID sporozoite markers (right). See Supplementary Table S1 online for gene descriptions of the first 30 UIS genes.

To date, there are no reports of *Plasmodium* sporozoites being individually isolated using droplet-based systems. We performed scRNA-seq using 10x Genomics’ droplet-based technology, selected for its ability to isolate thousands of cells in a relatively quick and cost-effective manner^29^. To minimize the risk of sequencing mosquito content, GFP *P. berghei* ANKA sporozoites dissected from the MIDs and SGs of *An. stephensi* mosquitoes were purified using a density gradient^30^ (Supplementary Fig. S1a online). Sporozoites from MIDs and SGs were then mixed together, before they were individually partitioned into reagent-containing microdroplets enabling the generation of cell-barcoded cDNA libraries. In order to assess the reproducibility of the technology and our workflow, we sequenced three mixed MID:SG sporozoite libraries derived from two different mosquito feeds (Fig. 1a; Supplementary Fig. S1b online).

Our single-cell libraries (Pb1, Pb2, Pb3) were of high-quality and majority of the reads (average of 59% across all three replicates (840,936,867/1,427,527,228)) aligned to the *P. berghei* ANKA genome (PlasmoDB, v46) (Fig. 1b; Supplementary Fig. S1d,e online). Of the reads aligning to the genome, 41% (341,097,547/840,936,867) mapped to regions encoding for *P. berghei* ANKA transcripts (Fig. 1b). After removing low-quality cells, we obtained transcriptomic profiles of 8,354 sporozoites (Fig. 1c), and detected 1,648 unique genes (~ 31% of the 5,245 known genes in the *P. berghei* ANKA genome) across all replicates (Fig. 1d). Pseudobulk analysis of the data revealed robust detection of highly abundant transcripts, with low-abundance transcripts serving as major contributors to detection variability across the three replicates (Supplementary Table S2 online). We found similar values across the three replicates upon assessing the median unique molecular identifiers (UMIs) per sporozoite (Pb1, 329; Pb2, 284; Pb3, 237) and unique transcripts per sporozoite (Pb1, 193; Pb2, 177; Pb3, 153) (Fig. 1e).

After independently assessing the sequenced libraries generated from the three single-cell captures, we integrated the three replicates by first identifying pairwise correspondences of highly variable genes between individual sporozoites, and then using this information to harmonize the datasets^31,32^. Low dimensional representation of the data, using a Uniform Manifold Approximation and Projection (UMAP), revealed two distinct populations of sporozoites (Fig. 1f). As depicted in the UMAP, we detected various “up-regulated in infective sporozoite” (UIS) genes, of which PBANKA_1328000 (Serine/threonine protein phosphatase; UIS2), PBANKA_1400800 (UIS3), PBANKA_0501200 (Early transcribed membrane protein; UIS4), and PBANKA_1128100 (Phospholipase, UIS10) were among the most highly expressed (Fig. 1g). We also found genes with known expression in MID sporozoites, such as PBANKA_0901300 (Membrane-associated erythrocyte binding-like protein) and PBANKA_1306500 (TRAP-like protein; UOS3) (Fig. 1g). These results are twofold: first, they validate our protocol for scRNA-seq analysis of sporozoites, and second, they show that high-throughput droplet-based scRNA-seq is a feasible method capable of assessing gene expression in *Plasmodium* sporozoites.

### Unsupervised graph-based clustering resolves sporozoite heterogeneity

**Figure 2:**
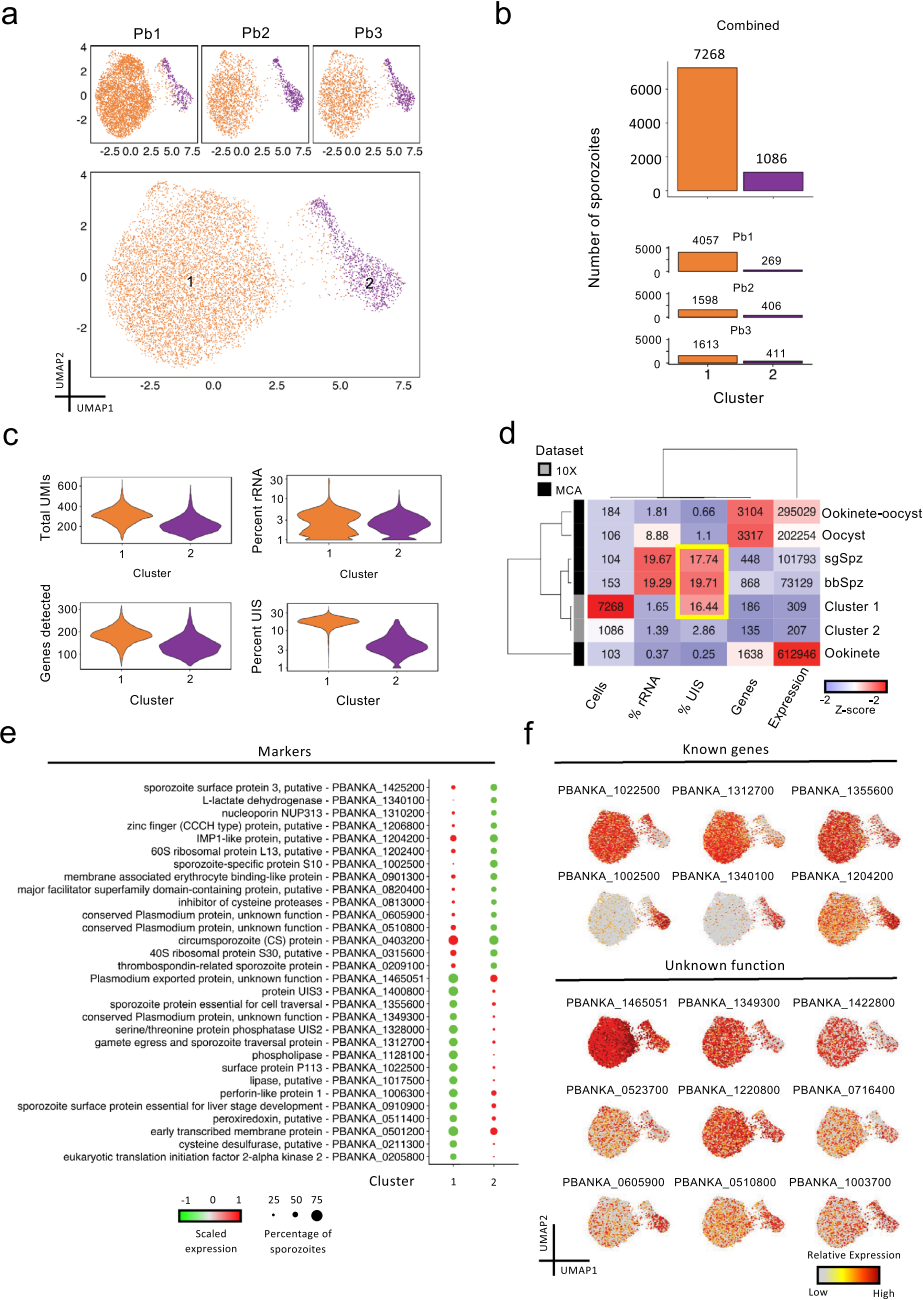
Unsupervised graph-based clustering of sporozoite 10 × scRNA-seq data. (**a**) UMAP plots displaying detected sporozoite populations in individual datasets (top) and overlaid (bottom). Algorithm used to cluster sporozoites = Leiden; resolution = 0.1. (**b**) Number of sporozoites in each of the resolved clusters. (**c**) Violin plots showing the distribution of total UMIs (top left), unique transcripts (bottom left), percentage rRNA counts for each sporozoite (top right), and percentage of UIS counts for each sporozoite (bottom right) across each of the clusters. Percentage was calculated by taking the sum of counts for indicated features belonging to the cluster divided by the sum for all genes multiplied by 100. (**d**) Heatmap indicating various metrics associated with sporozoite populations generated from the current dataset and MCA dataset. Values for cells column represent the total number of parasites analyzed; for % rRNA, % UIS, genes and expression columns, values represent median value per cell. (**e**) Dot plot illustrating top sporozoite markers in clusters 1 and 2. All plotted genes have an adjusted P value < 0.05; statistical significance was assessed by using Wilcoxon rank-sum test. See Supplementary Table S2 online for the complete list. (**f**) UMAP representations displaying top differentially expressed known (top) and unannotated (bottom) sporozoite markers.

In addition to the mapping of curated data on UMAP representations, unsupervised clustering is an effective and scalable approach in identifying cell populations. We performed graph-based clustering using the Leiden algorithm^33^ to further profile MID and SG sporozoites. Using a conservative resolution (Supplementary Fig. S2a online), we identified two distinct clusters, in all likelihood encoding for parasites derived from the two anatomical locations in the mosquito (Fig. 2a). Loading 7,500 SG sporozoites (Supplementary Fig. S1a online), the number in cluster 1 (7,268) approximates the number of cells we expected to obtain (Fig. 2b; Supplementary Fig. S2b online). Cluster 2, however, comprised 1,086 sporozoites, which was a much lower output than the 3,500 MID sporozoites we expected (Fig. 2b; Supplementary Fig. S2b online). We attributed this discrepancy to suboptimal processing of MID sporozoites from the second mosquito feed.

We then assessed global patterns of gene usage and expression in the two clusters alongside metrics obtained from the Malaria Cell Atlas (MCA)^17^ (Supplementary Fig. S2c,d online). Both the median number of transcripts per sporozoite—186 in cluster 1 and 135 in cluster 2—and the median expression per sporozoite—309 in cluster 1 and 207 in cluster 2—varied (Figs. 2c,d). We detected a higher percentage of reads encoding for UIS genes in SG sporozoites (cluster 1; 16.44%) relative to MID sporozoites (cluster 2; 2.66%) (Figs. 2c,d). The percentage of reads encoding UIS genes in our SG sporozoites were similar to the *P. berghei* sporozoite populations from the MCA (sgSpz 17.74% and bbSpz 19.71%), which indicates that despite differences in the total number of genes detected and expressed (Fig. 2d), the global assessment of UIS expression is comparable between the two single-cell capture technologies. Our data corroborate previous reports that SG sporozoites, compared to the parasite’s other life-stages in the mosquito, have increased expression of UIS genes^17,34–37^.

Next, we identified sporozoite markers using the Seurat function findMarkers. We defined a marker as a transcript detected in greater than 50% of cells, and differentially expressed (adjusted P value < 0.05). Consistent with its higher percentage of reads encoding for UIS genes, cluster 1 markers included many of the known UIS genes (Fig. 2e; Supplementary Table S3 online). Other well-described genes encoding for proteins important for sporozoite functionality, such as PBANKA_1022500 (Surface protein P113), PBANKA_1312700 (Gamete egress and sporozoite traversal protein) and PBANKA_1355600 (Sporozoite protein essential for cell traversal; SPECT1), displayed higher expression in this cluster than in cluster 2 (adjusted P value < 0.05; Figs. 2e,f; Supplementary Table S3 online). Of the markers in cluster 2, PBANKA_1002500 (Sporozoite-specific protein S10), was the most prominent. Previously shown to be upregulated in oocysts^38^, we detected the transcript in 77% (836/1,086) of sporozoites in cluster 2, compared to only 13% of sporozoites in cluster 1, in addition to being differentially expressed (average logFC 3.49; adjusted P value = 1.34 × 10^–215^) (Figs. 2e,f; Supplementary Table S2 online). Other notable markers identified in cluster 2 were PBANKA_1340100 (L-lactate dehydrogenase) and PBANKA_1204200 (IMP1-like protein, putative) (Figs. 2e,f; Supplementary Table S3 online). In total, of the 44 differentially expressed genes (DEGs), about 20% (9/44) encoded for genes with unknown function (Figs. 2e,f; Supplementary Table S3 online), indicating that other markers linked to sporozoite biology may exist.

### Integration of mixed sporozoite scRNA-seq data with Malaria Cell Atlas allows for fine-tuning of clusters

**Figure 3:**
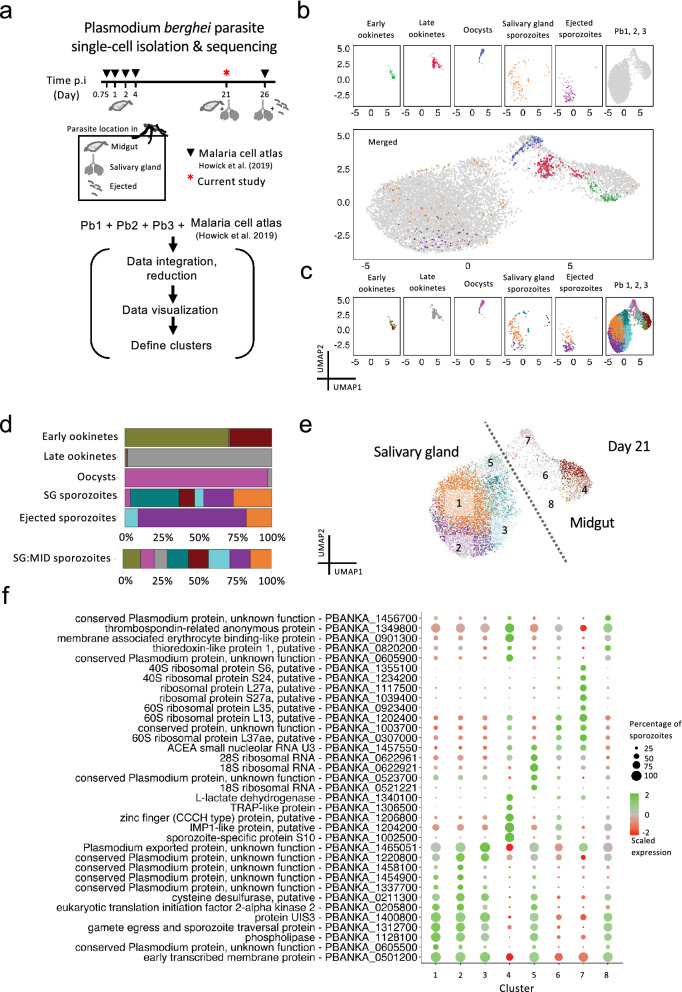
Redefining sporozoite populations using the Malaria Cell Atlas as a reference. (**a**) Sampling time points of *P. berghei* ANKA parasites at single-cell resolution during its development in the mosquito (top) and schematic illustrating the workflow used to redefine the clustering resolution for the mixed MID and SG sporozoite scRNA-seq data (bottom). (**b**) UMAP representations displaying integrated datasets colored by parasite life-stage individually (top) and overlaid (bottom). (**c**) UMAP representation displaying integrated datasets by cluster. (**d**) Distribution of parasites in each cluster across its different developmental stages. (**e**) UMAP representation displaying the redefined clustering of MID and SG sporozoites isolated on day 21 post mosquito infection. Dashed diagonal line represents the predicted break between SG and MID sporozoites. Algorithm used to cluster: Leiden, resolution = 0.7. (**f**) Dot plot illustrating top sporozoite markers in each of the clusters predicted to be composed of sporozoites derived from SGs and MIDs. All plotted genes have adjusted P value < 0.05; statistical significance was assessed by using Wilcoxon rank-sum test. See Supplementary Table S4 online for the complete list.

Cell annotation in single-cell datasets remains a challenging task, especially for species with limited or incomplete gene models such as *Plasmodium* species. A useful strategy for identifying unique cellular states is the integration of data with a single-cell reference atlas. We therefore integrated our sporozoite scRNA-seq dataset with the MCA’s collection of 650 single-cell transcriptomes of *P. berghei* parasites harvested from mosquitoes (Fig. 3a; Supplementary Fig. 3a online). UMAP reduction of the integrated datasets revealed overlap between the MCA’s day 26 sgSpz and bbSpz and our day 21 SG sporozoites (Fig. 3b, top and middle). These observed similarities indicate that despite the difference in harvesting time, the transcriptomic profiles of parasites are similar. A small population of MID sporozoites in our dataset overlapped with the transcriptomic profiles of ookinetes and oocysts harvested between 18 h and 4 days, but the majority had little in common with earlier developmental stages of the parasite (Fig. 3b, top and middle). This suggests that MID sporozoites harvested on day 21 have distinct transcriptional profiles compared to earlier stages of the parasite in this anatomical region of the mosquito.

Next, we used the graph-based clustering approach in Seurat to elucidate gene expression patterns across the various developmental stages. Unlike our mixed MID-SG sporozoite workflow, where the cellular origin of each transcriptome needed to be inferred, the plate-based workflow used by Howick and colleagues^17^ has the direct advantage of knowing the cellular origin of each transcriptome. Guided by the notion that a cluster should be composed primarily of parasites from a single-life stage, we used this information to discern a total of eight clusters present in our *P. berghei* sporozoite dataset (Figs. 3c,d; Supplementary Fig. S3b online). The majority of parasites in cluster 3 were day-21 SG sporozoites, while those in cluster 4 were mostly day-21 MID sporozoites (Figs. 3c,d,e). This suggests that the transcriptomic profiles of parasites at day 21 post-infection (p.i) are distinguishable from other developmental time points.

We then identified marker genes using the Seurat function FindAllMarkers, detecting many of the same markers as found with our aforementioned conservative clustering output (44 genes) (Supplementary Fig. S3c online), but also an additional 44 markers (Supplementary Fig. S3c online), including PBANKA_1306500 (TRAP-like protein), PBANKA_0619400 (V-type ATPase V0 subunit e, putative). There was considerable overlap of markers in clusters 1 and 2 (Fig. 3f; Supplementary Fig. S3d online; Supplementary Table S4 online), as well as modest changes in gene expression between them (Supplementary Table S5 online), suggesting slight heterogeneity amongst SG sporozoites at the gene expression level. Our fine-tuning had the largest benefit in resolving clusters 4 and 7, as the majority of their marker genes showed little co-occurrences of marker genes in other clusters (Supplementary Fig. S3d online).

In sum, the integration of our dataset with the MCA data revealed stage-specific gene expression patterns in *P. berghei* parasites as they develop in separate parts of the mosquito. This, in turn, provided us with a unique opportunity to refine our clustering strategy to resolve sporozoite sub-populations that we were unable to infer through an unsupervised clustering approach alone.

### Trajectory analysis reveals gene expression changes associated with sporozoite development

One caveat of analyzing a developmental system using a cluster-based classification strategy is that inclusion thresholds may artificially assign cells to groups when in fact cell transitions may be occurring in a more continuous manner. The co-occurrence of many marker genes across the eight clusters (Supplementary Fig. S3d online) suggests that a continuum of sporozoite transcriptional states may exist, as opposed to distinct states.

**Figure 4:**
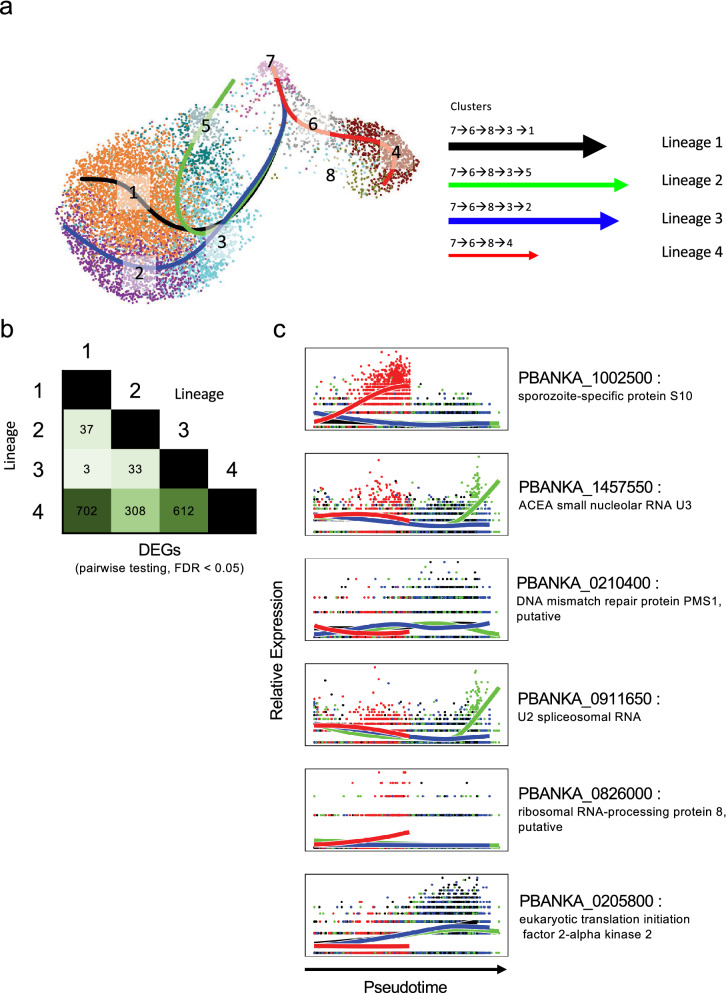
Pseudotime analysis of SG and MID sporozoite markers. (**a**) UMAP representation of SG and MID sporozoites clusters overlaid with the four principal curves identified using Slingshot to reveal potential developmental trajectories (lineages). Arrow width represents the relative number of cells in each lineage; length of arrows represents the length of trajectories for each lineage. (**b**) Number of DEGs when performing pairwise tests between each of the lineages. (**c**) Smoothed expression across the differentiation trajectories in SG and MID sporozoites for top genes changing across pseudotime. See Supplementary Table S6 for the complete list.

To study this further, we sought to identify changes in gene expression across pseudotime. Four developmental trajectories (lineages) from our MID and SG sporozoites were predicted using Slingshot^39^ (Fig. 4a). All of them traversed clusters 7, 6, and 8, suggesting shared gene expression dynamics prior to their divergence. Three out of the four trajectories terminated in a SG sporozoite population (clusters 2, 3, and 5), with the fourth ending in cluster 4, previously identified as a MID sporozoite population (Fig. 4a).

Next, we assessed DEGs between the trajectories using tradeSeq^40^, detecting a total of 661 genes with differential expression across pseudotime (FDR < 0.05) (Supplementary Table S6 online). The number of DEGs detected when each of the first three trajectories were compared to the fourth confirmed distinct expression patterns between SG and MID developmental trajectories (Figs. 4b,c; Supplementary Figs. S4a,b online; Supplementary Table S6 online). This data reveals variations in gene expression over pseudotime, and indicates that various developmental trajectories can be detected in sporozoites harvested at a single time point.

### Transcriptomic profiles of mature salivary gland and midgut sporozoites encode for different biological processes

**Figure 5:**
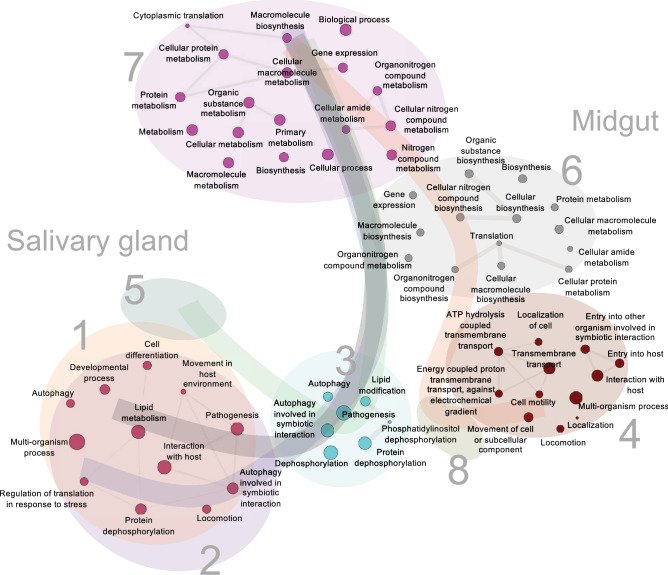
Enrichment analysis of SG and MID sporozoite markers. GO network analysis of enriched Biological Processes in sporozoite clusters. For clusters 1 and 2, markers co-occuring in these clusters were used to identify enriched BPs and the GO network analysis. Markers with an adjusted P value < 0.05 were used for gene set enrichment analysis. No significant enrichments were detected in cluster 5 and 8 using the current thresholds.

To make sense of the broader landscape of biological functions associated with the eight sporozoite clusters, we assessed the enrichment of various gene sets from the Gene Ontology (GO) database. Using the markers identified in each cluster as inputs (Supplementary Table S4 online), we identified 91 gene sets curated under “biological processes” in the GO database as significantly enriched (adjusted P value < 0.05; Supplementary Table S7 online). Due to the limited number of marker genes in clusters 5 and 8 (Supplementary Table S4 online), no enrichment was detected. Clusters 6 and 7 showed enriched pathways associated with translation (Supplementary Table S7 online). Assessment of gene sets derived from the markers found in clusters 1 and 2 indicated that they were functionally similar (Supplementary Figs. S5a,b online; Supplementary Table S7 online). They displayed considerable overlap in significantly enriched processes, such as exit from the host, movement within the host environment, and pathogenesis. Interestingly, cluster 3 exhibited much overlap with clusters 1 and 2, but was ultimately unique since many processes associated with post-translational modifications were also identified (Supplementary Fig. S5b online; Supplementary Table S7 online). Of the clusters composed of MID sporozoites, cluster 4 displayed distinct enriched pathways associated with energy production and locomotion, including ATP hydrolysis-coupled transmembrane transport, cell gliding, and entry into host (Supplementary Fig. S5b online; Supplementary Table S7 online). While this particular analysis remains limited due to the high number of uncharacterized genes in the *P. berghei* ANKA genome; as well as the broad nature of GO curation of known genes, it further supports heterogeneity amongst SG and MID sporozoites (Fig. 5).

## Discussion

To date, scRNA-seq studies on *Plasmodium* sporozoites have been based on data generated from low-throughput plate-based methods^17,20^, and the feasibility and utility of a high-throughput droplet-based scRNA-seq workflow of *Plasmodium* sporozoites have not yet been assessed. In this study, we show that 10x Genomics’ droplet-based scRNA-seq technology can effectively generate libraries for downstream genome-wide transcriptomic analyses of *P. berghei* sporozoites. Our analysis corroborates findings from previous transcriptomic studies in *P. berghei* sporozoites^17,35,37,41^ and provides new insights into heterogeneity at this developmental stage of the life-cycle.

Studying sporozoite biology at the molecular level is no easy task: in order to perform -omic analyses, researchers must obtain sufficiently pure samples to avoid the capture and amplification of mosquito material during the preparation of sequencing libraries. This requirement holds true for scRNA-seq efforts. We show that post-mosquito dissection, a discontinuous gradient purification procedure^30^ is effective in meeting the purity requirements necessary for the generation and sequencing of sporozoite single-cell libraries. Despite using this protocol and sufficient genome mapping, we found a significant proportion of genome-mapped reads mapping outside of the *P. berghei* ANKA transcriptome, particularly in regions adjacent to current gene boundaries. This is unsurprising, as the 3′ untranslated regions (UTRs) are poorly annotated in the gene models of *P. berghei* ANKA. At present, this is one limitation of 3′ poly-A selection single-cell approaches—such as 10x Genomics’ technology—in *Plasmodium* species (like any other poorly annotated species), as reads mapping to the 3′ UTR of a transcript may not be assigned and counted. With the increased availability of refined gene models^42,43^, we expect these updates to help quantification efforts, which will ultimately provide a clearer picture of gene usage in *Plasmodium* parasites.

Droplet-based scRNA-seq studies are often performed in individual replicates (i.e. libraries generated in a contained workflow), due to both cost and sample limitations, despite the fact that technical variation can affect preparations. Here, we generated scRNA-seq libraries—the first to benchmark 10x Genomics’ droplet-based single-cell gene expression technology in *Plasmodium* sporozoites—from three separate replicates to assess sensitivity and run-to-run variability of the technology. Overall, we found good reproducibility between the three sporozoite libraries generated across the two mosquito feeds, with each having similar per-cell metrics. It is important to keep in mind that the sporozoites assessed in this study were derived from a lab-adapted *Plasmodium* parasite model^44^. Whether these similarities in per-cell metrics can be extrapolated to sporozoites from field isolates remains to be determined.

In *P. berghei*, various forms of gene regulatory mechanisms have been shown to occur at key transition stages of the parasites life cycle^45,46^. Here, although its protein levels cannot be inferred, the detection of *puf2* transcripts (PBANKA_0719200) and its slightly increased expression in SG populations is consistent with previous reports highlighting the protein’s role in translational repression of mRNA. Interestingly, recent single-cell transcriptomic profiling of *Plasmodium* parasites reveals that relative to other stages of the parasite’s life cycle, the number of transcripts detected in sporozoites is lower when compared across like technologies^17,20^. The low number of unique transcripts detected in MID and SG sporozoites in our study is consistent with these reports. Together, these observations suggest that gene repression may serve as an additional layer of regulation in sporozoites. Future studies to identify key transcriptional regulators that may be underlying these observations are warranted.

We mixed SG and MID sporozoites within each of our replicates for two reasons: first, so they could be treated without confounding each other, and second, to test whether data reduction and visualization techniques could effectively separate sporozoites harvested from two different anatomical sites of a mosquito on the same developmental day. To distinguish between SG and MID sporozoites, we used previously-reported data indicating a strong relationship between UIS expression and sporozoite development^5,6,34,48^. To identify additional markers linked to sporozoites’ developmental status, we used an unsupervised graph-based clustering approach, assigning sporozoites to a cluster based on their gene expression profiles. One of the major challenges of scRNA-seq is identifying cell states at a particular level of resolution^49^. Although unsupervised graph-based clustering does not rely on a priori information to guide its output, choosing a meaningful resolution remains a challenge. With this in mind, we started with a conservative clustering resolution that allowed for the comparison of sporozoites based on the anatomical site that they were harvested from. Differential gene expression analysis between the two broadly defined MID and SG populations, in turn, allowed for the identification of additional markers linked to the sporozoites’ anatomical origin. Many of the markers we detected were conserved proteins with unknown function in *P. berghei*, suggesting the existence of genes linked to sporozoite infectivity, immunogenicity, and motility that have yet to be explored.

To fully exploit our single-cell data, and to paint a better picture of the parasites’ development in the mosquito, we integrated our dataset with the *P. berghei* scRNA-seq data from the MCA^17^. As sporozoites are found in substantial numbers inside SG of mosquitoes after twelve days post-feeding^50^, we expected sporozoites harvested from the SGs 21 days post-feeding to have somewhat similar transcriptional profiles to those harvested on day 26. We confirmed this expectation upon observing significant overlap between the two datasets. Parasites derived from earlier developmental time points (18–48-h ookinetes and day 4 oocysts) were clearly distinguishable from our day 21 SG sporozoites. Furthermore, the slight overlap of these earlier stages with our day 21 MID sporozoites highlights the unique gene usage of sporozoites prior to SG invasion. Interestingly, a portion of the day-21 SG sporozoites overlapped with the day-26 bbSpz, suggesting that some SG sporozoites have an “activated” phenotype before their ejection from the mosquito. A similar finding in scRNA-seq data from *P. falciparum* SG sporozoites has recently been reported^20^. Although the possible activation of parasites during the handling process cannot be ruled out, these observations may provide clues to the molecular underpinnings associated with the sporozoite’s location in the SG^51^ or its capacity to successfully invade its host^52,53^.

To further assess sporozoite heterogeneity at the transcriptional level, we re-performed the clustering analysis using the various parasite transcriptomes from the MCA dataset as a guide. Given that the stage of the parasite was known in the MCA, we reasoned that transcriptomes derived from parasites at the different developmental time points inside the mosquito should each make up their own cluster. We then re-classified our previously identified sporozoite clusters, branching out from two clusters to eight, which allowed us to infer subpopulations of SG and MID sporozoites, as well as to identify additional markers associated with development.

Trajectory analysis is a valuable tool in deciphering genes important in the transition from one cellular state to the next. Applied to sporozoites, we inferred various developmental paths, and the underlying changes in gene expression of markers as the sporozoite differentiates. We detected 661 differential expressed genes over pseudotime (adjusted P value < 0.05), supporting the notion that sporozoite transitions may occur in a more continuous manner. Further work is required to better understand the factors that could be involved in shaping these developmental trajectories, and whether similar patterns exist in sporozoites derived from other *Plasmodium* species.

To date, only 54% of the genes in *P. berghei* ANKA are annotated for involvement in a given biological process. Of the genes that are annotated, many are described using GO terms encoding for broad cellular processes. These limitations make it difficult to fully appreciate the biological roles of genes, and their interaction in complex networks in the parasite. Despite these limitations, GO enrichment analysis applied to our dataset allowed for the identification of functionally related genes in MID and SG sporozoites, and provided a systems-level perspective of gene regulatory programs across sporozoite populations.

The ability to measure gene expression in thousands of individual sporozoites provides a new means of assessing heterogeneity, and may provide clues to mechanisms underlying mosquito-parasite interactions and parasitic invasion, whether of the mosquito SG or the mammalian host. Our assessment of day 21 sporozoites from two anatomical positions in the mosquito provides new insights into the RNA landscapes of mature MID and SG sporozoites at single-cell resolution. It has been shown previously that as early as day 12, *P. berghei* sporozoites begin to enter the SGs of mosquitos^50^. At day 21, some sporozoites may begin to degenerate. The transcriptomic signature of a degenerating sporozoite remains unknown and this gap in knowledge may limit the interpretation of the predicted developmental trajectories presented in this study. Furthermore, given our analysis we cannot be sure as to why the MID sporozoites still reside in this anatomical location or if they will eventually invade the SGs. Whether there is a causative link between the transcriptomic states of these MID sporozoites and their infectivity status is unknown. These uncertainties notwithstanding, our work offers an interesting snapshot of the transcriptomic states of mature MID sporozoites at day 21 and we hope that our analyses prompt more targeted, gene-specific studies to better understand the biological significance of these sporozoites.

Future scRNA-seq studies on *Plasmodium* parasites will benefit from the availability of both high-throughput droplet-based methods and high-coverage plate-based methods. In addition, as the number of single-cell datasets grows, researchers will have the opportunity to perform inter-species comparisons to better understand parasite heterogeneity and gene expression dynamics. We hope that our *P. berghei* sporozoite scRNA-seq data serves as a valuable resource for the malaria research community.

## Material and methods

### Ethics statement

All animal experiments were approved by the Animal Care and Use Committee of Institut Pasteur (CETEA Institut Pasteur 2013-0093, Ministère de l’Enseignement Supérieur et de la Recherche MESR 01324) and were performed in accordance with European guidelines and regulations (directive 2010/63/EU).

### Mice, parasites, mosquitoes, and infections

Female Swiss mice purchased from Javier Labs were housed under 12 h:12 h light:dark conditions and with ad libitum access to food and water. Mice were inoculated via intraperitoneal injection with 500µLof rat blood infected with *P. berghei* ANKA clones expressing GFP, under the control of the *hsp70* regulatory regions^44^. On day 3 after infection, parasitemia was determined from the mice by FACS and blood-smear counting. *Anopheles stephensi* mosquitoes (SDA500 strain) reared in the Centre for Production and Infection of Anopheles (CEPIA) at the Institut Pasteur (Paris, France) were fed on infected mice in the manner described previously^54^.

### Sporozoite isolation

Sporozoites were isolated from MIDs and SGs of *An. stephensi* 21 days after an infectious blood-meal. Mosquito infectivity was confirmed by the presence of GFP-expressing sporozoites in both SGs and MIDs (Supplementary Fig. 1a online). MIDs and SGs from infected mosquitoes were dissected under a stereozoom microscope and placed in separate microcentrifuge tubes containing 50 μL of ice-cold PBS. The number of dissected mosquitoes was variable depending on the efficiency of sporozoite production. Overall, between 50 and 80 mosquitoes were dissected for each sample. After the release of sporozoites via manual disruption, sporozoites were purified using a discontinuous density gradient protocol adapted from Kennedy and colleagues^30^. Briefly, 450µL of HBSS containing phenol-red was added to the 50 μL PBS solution containing sporozoites and mosquito content. Mixtures containing mosquito content and sporozoites were placed on 3 mL of a 17% Nycodenz (Axis-Shield, Norway) solution and spun at 2500 g for 20 min in a centrifuge pre-chilled to 4 °C with no brake. Next, 300 μL of purified sporozoites were carefully removed from the interface and spun at top speed in a 4 °C centrifuge for 10 min to pellet the sporozoites. The supernatant was removed to maintain around 50uL of purified sporozoites. Resuspended sporozoites were then passed through a 20 µm mesh into a microcentrifuge tube and held on ice until further processing.

### Single-cell library preparation and sequencing

Post purification and counting, SG and MID sporozoites were pooled in either 50:50 or 90:10 SG:MID ratios. From the 50:50 SG:MID sporozoites preparation, we loaded equal amounts of sporozoites onto two wells of a Chromium Chip B, giving a total of three sporozoite mixtures derived from two separate mosquito feeds (Supplementary Fig. 1b online). Post gel beads-in-emulsion (GEM) generation, single-cell libraries were processed according to the 10 × Chromium 3′ v2 User Guide protocol with some modifications. First, due to the low RNA content of *Plasmodium* species, following GEM-RT incubation and cleanup, we used 14 PCR cycles to amplify the full-length cDNA to ensure the generation of sufficient mass for library construction. Second, during the sample index PCR step, we used 16 cycles. Prepared single‐cell libraries were sent to Novogene (Hong Kong) or Macrogen (Seoul, South Korea) for sequencing using an Illumina HiSeq Xten sequencer in stand-alone mode with the following parameters: 26 cycles (read 1), 8 cycles (i7 index), and 98 cycles (read 2).

### Alignment, cellular barcode assignment, and gene quantification

Quality of RNA-sequencing libraries was assessed using FASTQC^55^. The *P. berghei* ANKA genome (v46) and its corresponding genomic features file (GFF) were downloaded from PlasmoDB.org. The GFF was converted to GTF format, then using the genome and GTF, we generated a genome index in STAR^56^ (v2.3.7a) using the –runMode genomeGenerate specifying the following additional parameters: –genomeSAindexNbases 11 –sjdbOverhang 97. Next, we downloaded the V2 barcode whitelist from the 10x Genomics’ website. Mapping, demultiplexing and gene quantification was performed using STAR’s turnkey solution for analyzing droplet-based scRNA-seq sequencing data by specifying the following options: –soloType CB_UMI_Simple –soloCBlen 16 –soloUMIlen 12 –soloCBwhitelist */path/to/10X/V2/whitelist* –alignIntronMin 1 –alignIntronMax 2500 –outFilterType BySJout –outFilterIntronMotifs RemoveNoncanonical –soloCBmatchWLtype 1MM_multi_pseudocounts –soloUMIfiltering MultiGeneUMI –soloUMIdedup 1MM_All –soloFeatures Gene. The alignIntronMin and alignIntronMax parameters were set based on the minimum and maximum intron lengths of all annotated mRNA transcripts calculated upon generation of the indexed genome (Supplementary Fig. S5a,b online). The resulting unfiltered (raw) matrix, features, and barcodes files for each sample were used for further processing in R (version 4.2). A schematic of the workflow and sequencing statistics for each sample is shown in Supplementary Fig. 1c online.

### Filtering and normalization

To filter out empty droplets and retain GEMs containing sporozoites, we used the emptyDrops function^57^ with an FDR cutoff of 0.001. Next, to account for multiple sporozoites captured in the same droplet, sporozoites with counts greater than 3 deviations from the median (MAD) were removed. After removing low-quality cells, we removed genes with low detection; keeping genes with greater than two counts in at least two cells. See Supplementary Table S8 for a full breakdown of loading, alignment, and per-cell metrics.

### Integration of scRNA-seq libraries

Filtered count matrices from the three replicates (Pb1, Pb2, Pb3) were transformed into a Seurat (v3.1.0) object and processing was performed using functions coming from the Seurat package in R. Prior to integration, each dataset was normalized using the LogNormalize function whereby gene counts for each cell were divided by its total counts and multiplied by a scale factor of 1000. Values were natural-log transformed using log1p. Highly variable features were identified for each replicate using the FindVariableFeatures function with the following parameters provided: selection.method = "vst", nfeatures = (total transcripts) ✕ 0.2. Next, integration anchors were found using the FindIntegrationAnchors function with the following parameters: dims = 1:15, anchor.features = 300. Using these anchors, the datasets were integrated using the IntegrateData function.

### Dimension reduction and clustering

Following integration, data was scaled and dimensionality reduction was performed using principal component analysis (PCA) and UMAP. Next, an unsupervised graph-based clustering approach was used to identify sporozoite communities. First, k-nearest neighbors were found and a shared nearest neighbor (SNN) graph was constructed using the Seurat function FindNeighbours with the following parameters: reduction = "pca", dims = 1:15. After the optimization of the SNN modularity, clusters of cells were identified using Seurat’s FindClusters function with the Leiden algorithm^33^ selected. Clustering outputs were assessed at various resolutions and cluster stability was visualized using a Clustering tree plot^58^.

### Integration of 10x scRNA-seq data with Malaria Cell Atlas scRNA-seq data

The MCA’s aligned and counted dataset was cloned from GitHub (https://github.com/vhowick/MalariaCellAtlas/tree/master/Smartseq2analysis/PCA_SS2). All 1787 cells across various stages of the *P. berghei* parasite’s life cycle and associated gene expression counts were imported into R. We next subsetted the parasites based on their developmental status, keeping only stages resident in the invertebrate host. Cell and gene filtering were performed in the same manner described above. The *P. berghei* sporozoite datasets generated in this study (Pb1, Pb2, Pb3) and subsetted MCA dataset were then assembled into an integrated reference using the method described in “Integration of scRNA-seq libraries”.

### Differential gene expression analysis

To detect cluster-specific markers, the Seurat functions FindAllMarkers or FindMarkers were used. Only transcipts in more than 50% of cells in a cluster were considered. All logFC values were considered and comparisons with adjusted P values < 0.05 were considered significant.

### Trajectory analysis

To resolve sporozoite lineages from the scRNA-seq data, we used the UMAP embeddings, clusters (eight), and variable feature counts from our sporozoite dataset after refinement with the MCA, as input for pseudotime analysis. Lineages and smoothed curves were generated using Slingshot^39^ with the default parameters selected. Next, to identify genes with altered expression across pseudotime a negative binomial generalized additive model (GAM) for each gene was generated using the tradeSeq^40^ function fitGAM. Based on the fitted models, we used the tradeSeq function diffEndTest to identify genes that were differentially expressed between lineages. Briefly, the function performs a global test, with the null hypothesis that the average expression at the endpoints is equal for all lineages using a multivariate Wald test. In addition to the global testing, we performed pairwise comparisons between lineages.

### Gene set enrichment analysis

Marker genes (adjusted P value < 0.05) predicted from each cluster were uploaded to PlasmoDB and gene set enrichment analysis was performed using the built-in Gene Ontology tool with the following parameters indicated: Organism—*Plasmodium berghei* ANKA; Ontology—Biological Process; Evidence—Computed, Curated; Limit to Go Slim terms—No; P-Value cutoff— 0.05. Enriched gene sets were exported to REVIGO^59^ to reduce redundant GO terms using the default parameters and with the GO term database ‘*Plasmodium falciparum*’ (closest relative to *Plasmodium berghei* in the database) selected. Resulting lists were uploaded to Cytoscape^60^ for graph-based representations of enriched pathways in each cluster.

## Supplementary Information

Supplementary Information 1.

Supplementary Tables.

## Data Availability

All raw sequencing data have been deposited in the European Nucleotide Archive at European Molecular Biology Laboratory European Bioinformatics Institute (www.ebi.ac.uk/ena/) under accession number ERP123892. Scripts and supporting files are available on GitHub at: https://github.com/AnthonyRuberto/Pb_Spz_singleCell. Archived scripts and output files as at time of publication are available on Zenodo at https://doi.org/10.5281/zenodo.4165032
