# Supplementary material for: Single-cell RNA sequencing reveals developmental heterogeneity among Plasmodium berghei sporozoites
Source: Sci Rep. 2021 Feb 22;11:4127. doi: 10.1038/s41598-021-82914-w (PMC7900125; doi:10.1038/s41598-021-82914-w)

Single-cell RNA sequencing reveals developmental heterogeneity among *Plasmodium berghei* sporozoites

Anthony A. Ruberto[1*,] Caitlin Bourke[2,3], Nicolas Merienne[1], Thomas Obadia[1,4], Rogerio Amino[1], Ivo Mueller[1,2,3*]

[1] Department of Parasites and Insect Vectors, Institut Pasteur, Paris, France

[2] Division of Population Health and Immunity, Walter and Eliza Hall Institute of Medical Research, Parkville, Victoria, Australia

[3] Department of Medical Biology, University of Melbourne, Melbourne, Victoria, Australia

[4] Hub de Bioinformatique et Biostatistique – Département Biologie Computationnelle, Institut Pasteur, 75015 Paris, France

* Correspondence and requests for materials should be addressed to A.A.R (email: aruberto@pasteur.fr) or I.M. (email: mueller@wehi.edu.au)

Supplementary figures S1-6

Supplementary Figure 1. Related to Figure 1. Mapping, quality control, and integration of *P. berghei* sporozoite 10x scRNA-seq data.
(a) Representative images of an *Anopheles stephensi* mosquito infected with GFP-expressing *P. berghei* ANKA 21 days after an infectious blood meal (left) and GFP-expressing *P. berghei* ANKA sporozoites post purification (right).
(b) Detailed schematic illustrating the workflow used to generate *P. berghei* ANKA scRNA-seq data.
(c) Detailed schematic illustrating the steps and parameters used to align scRNA-seq reads to the *P. berghei* ANKA genome to obtain individual sporozoite transcriptomes.
(d) Total number of reads aligning to *P. berghei* ANKA and *An. stephensi* genomes in each of the three replicates.
(e) Quality metrics of reads mapped to the *P. berghei* ANKA genome in each replicate.

Supplementary Figure 2. Related to Figure 2. Unsupervised graph-based clustering of sporozoite 10x scRNA-seq data.
(a) Clustering tree showing the relationship between clusters (left) and the corresponding UMAP representations of *Pb* 1,2,3 datasets integrated (right) at various resolutions. Red arrow indicates the clustering resolution chosen for the analysis in Figure 2. Clustering tree was generated using the Clustree package[58] in R.
(b) Proportion of cells in each of the clusters across the three datasets using the resolution (0.2) chosen for the analysis in Figure 2.
(c) Number of MCA single-cell parasite transcriptomes analyzed.
(d) Violin plots showing the distribution of gene counts (top left), unique transcripts (bottom left), percentage rRNA counts for each sporozoite (top right), and percentage of UIS counts for each sporozoite (bottom right) across various the life stages of *P. berghei* in the mosquito. MCA single-cell data was obtained from Howick et al.[17]. Percentage was calculated by taking the sum of counts for indicated features belonging to the life-stage divided by the sum for all genes multiplied by 100. Horizontal dashed line = 20%.

Supplementary Figure 3. Related to Figure 3. Redefining sporozoite populations using the Malaria Cell Atlas as a reference.
(a) UMAP representation of scRNA-seq *P. berghei* ANKA datasets colored by the parasites life stage. Data obtained from Howick et al.[17] was re-analyzed using the analysis pipeline described in the methods.
(b) Clustering tree showing the relationship between clusters at a range of resolutions (left) and the corresponding UMAP representations of Pb 1,2,3 and MCA datasets integrated (right). Red arrow indicates the clustering resolution chosen for the analysis in Figure 3. Clustering tree was generated using the Clustree package[58] in R.
(c) Venn diagram displaying the number of unique and overlapping markers detected when using clustering strategy using graph-based strategy alone (2 clusters) and graph-based clustering strategy guided by MCA integration (8 clusters).
(d) Upset plot showing unique and co-occurring markers across the eight clusters. Inlet: Venn diagram displaying the number of overlapping and unique markers when comparing clusters 1 and 2.

Supplementary Figure 4. Related to Figure 4. Pseudotime analysis of SG and MID sporozoite markers.
(a) Smoothed expression across the differentiation trajectories in SG and MID sporozoites for select UIS and UOS markers.
(b) Smoothed expression across the differentiation trajectories in SG and MID sporozoites for the nine *P. berghei* genes with unknown function identified as differentially expressed using a clustering resolution of 0.1.

Supplementary Figure 5. Related to Figure 5. Enrichment analysis of SG and MID sporozoite markers.
(a) GO network analysis of enriched Biological Processes in clusters 1 and 2 when performed separately. Markers with an adjusted P value < 0.05 were used for gene set enrichment analysis.
(b) Upset plot displaying the co-occurrence of enriched GO biological gene sets across the 8 clusters

Supplementary Figure 6. Related to Material and Methods. Alignment, cellular barcode assignment, and gene quantification.
(a) Number of annotated genes, RNA species, and mRNA exons and introns in the *P. berghei* genome (PlasmoDB, v46).
(b) Distribution of the lengths (# of nucleotides) of genes, RNA species, and mRNA introns and exons in the *P. berghei* genome (PlasmoDB, v46).

Ruberto *et al*. Supp. Fig. S1

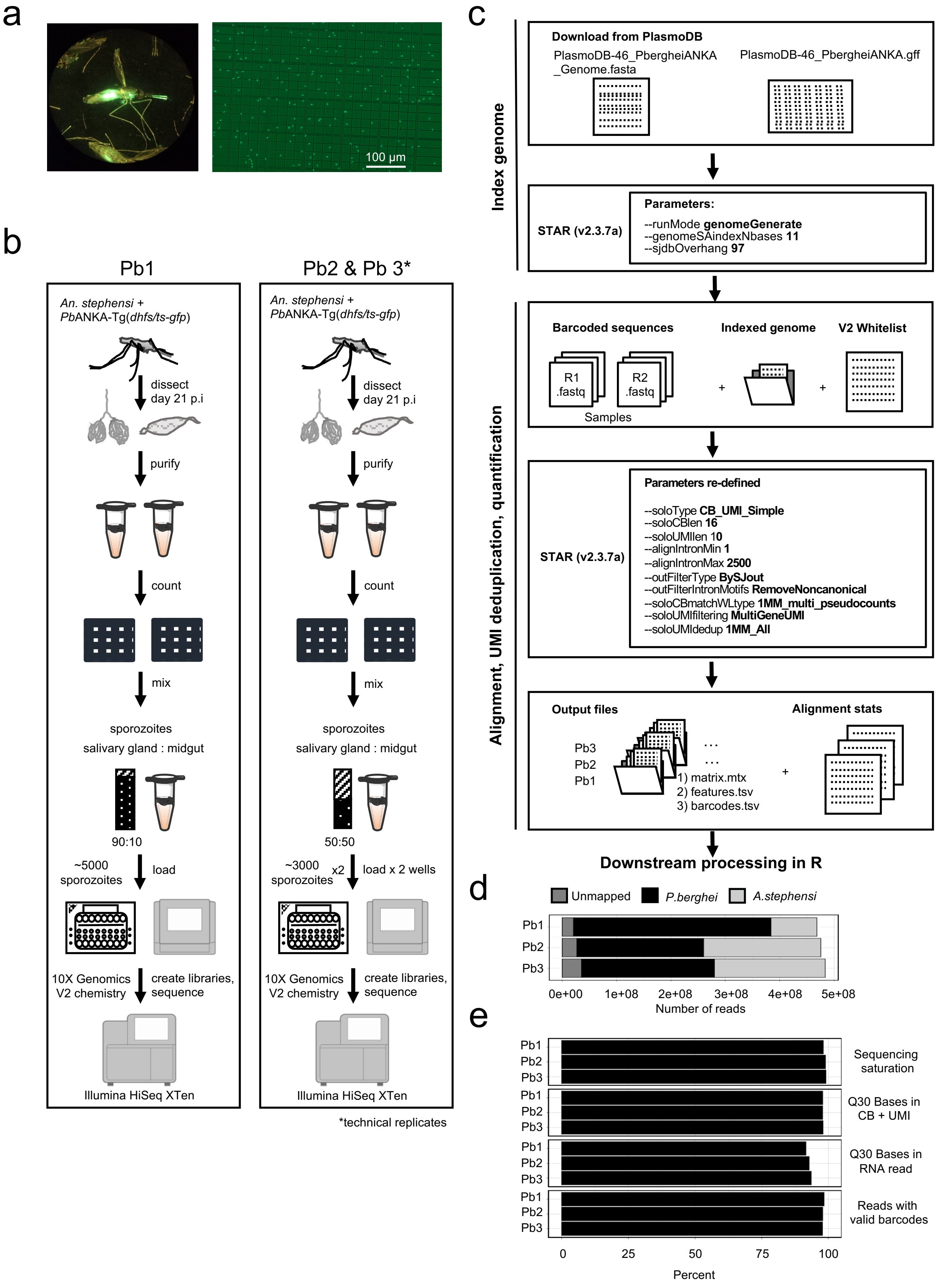

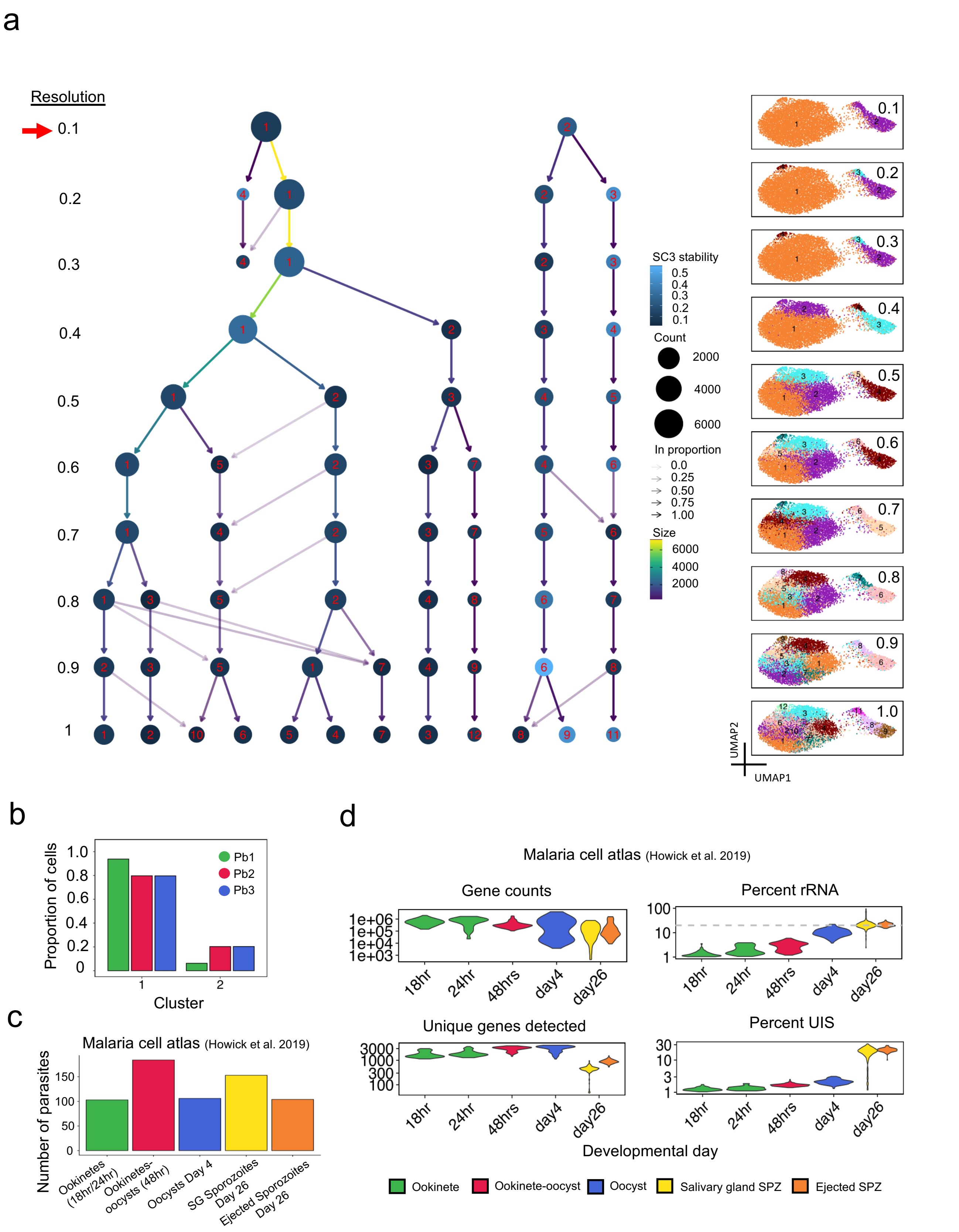
a
Resolution
0.1
0.2
0.3
0.4
0.5
0.6
0.7
0.8
0.9
1
SC3 stability
Count
2000
4000
6000
In proportion
0.0
0.25
0.50
0.75
1.00
Size
6000
4000
2000
UMAP2
UMAP1
b
Proportion of cells
Cluster
Pb1
Pb2
Pb3
c
Malaria cell atlas (Howick et al. 2019)
Number of parasites
Ookinetes (18hr/24hr)
Ookinetes-oocysts (48hr)
Oocysts Day 4
SG Sporozoites Day 26
Ejected Sporozoites Day 26
d
Malaria cell atlas (Howick et al. 2019)
Gene counts
Percent rRNA
Unique genes detected
Percent UIS
18hr
24hr
48hrs
day4
day26
Developmental day
Ookinete
Ookinete-oocyst
Oocyst
Salivary gland SPZ
Ejected SPZ

Ruberto *et al*. Supp. Fig. S3

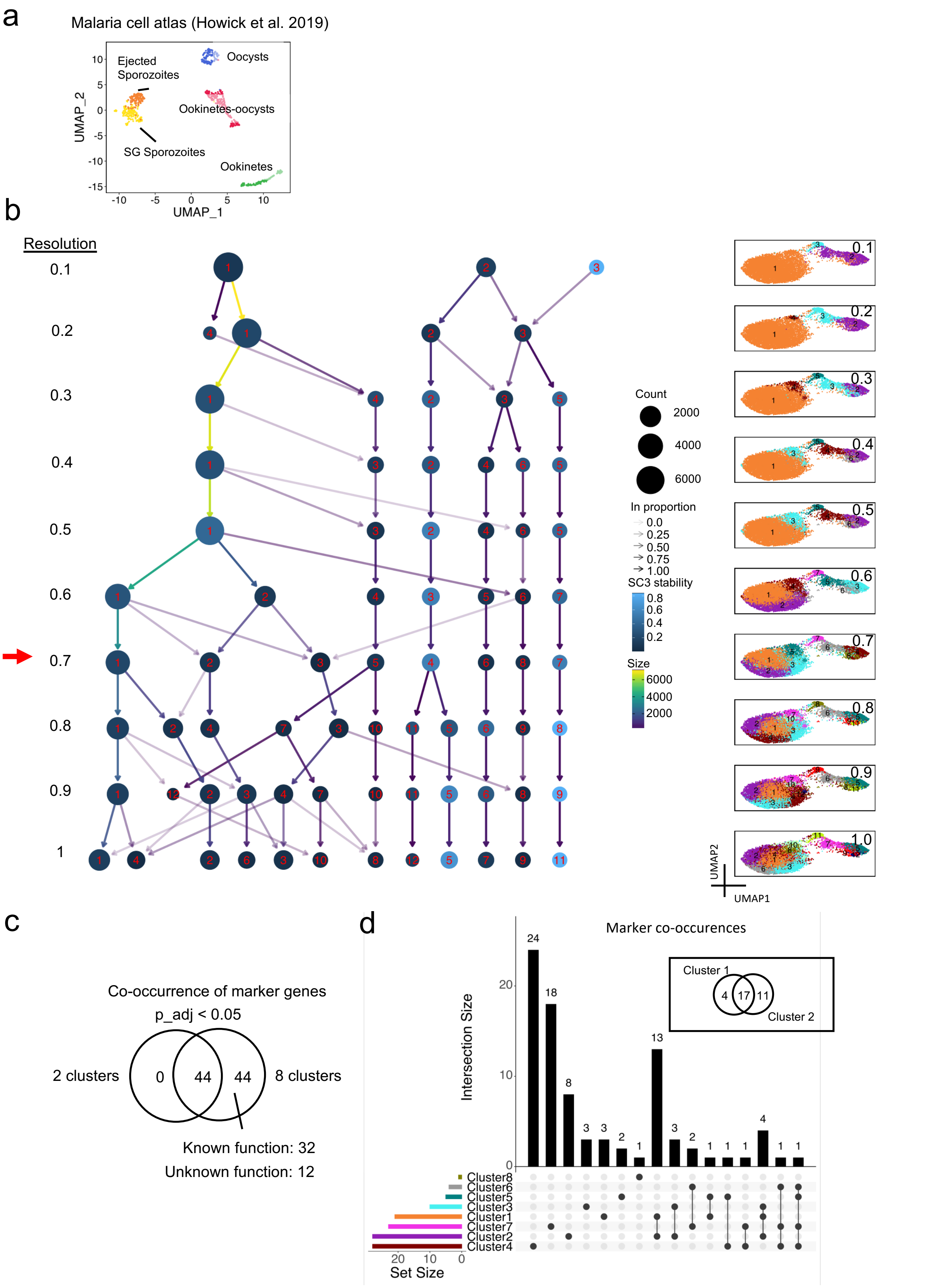

Ruberto *et al*. Supp. Fig. S4

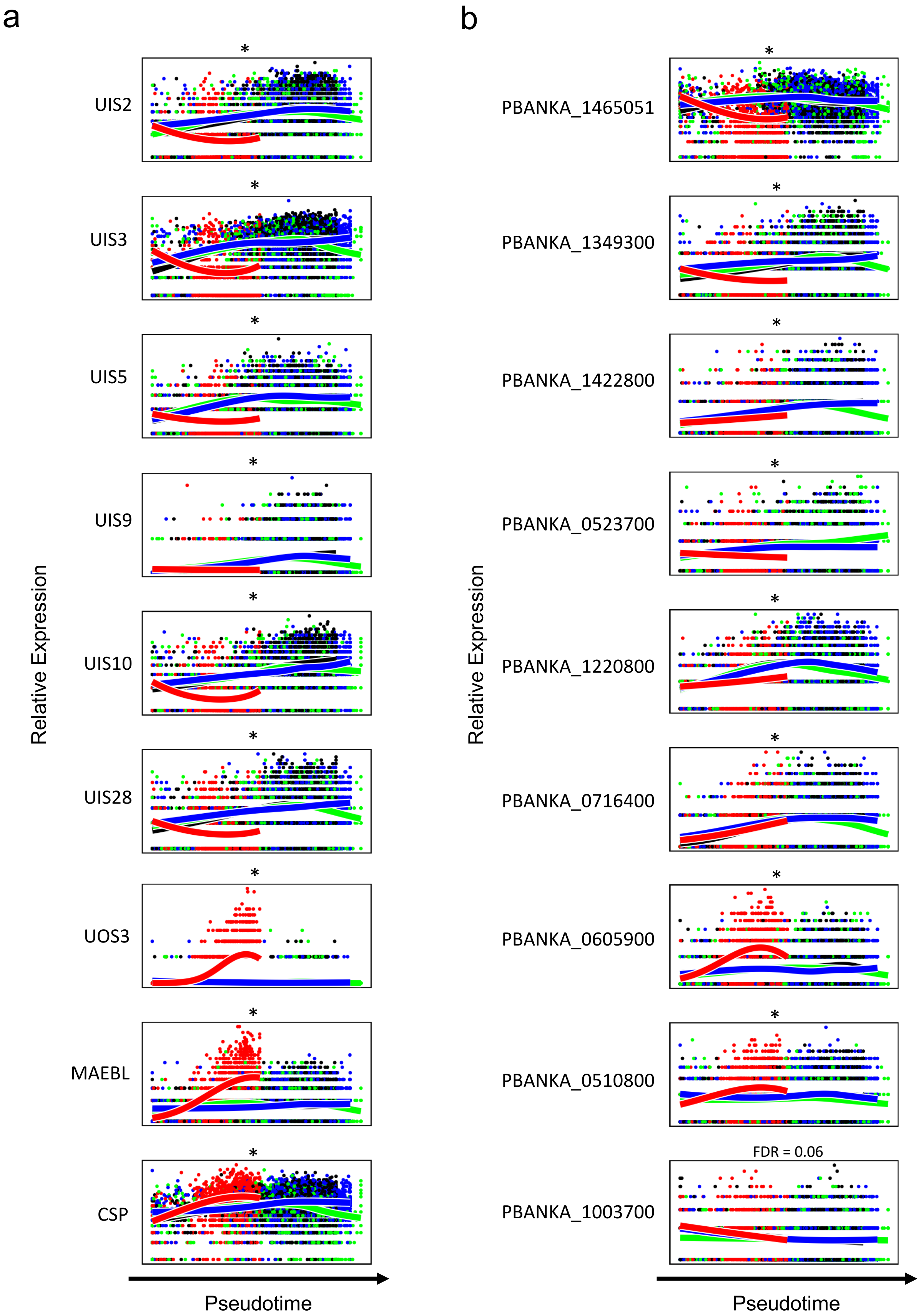

* FDR < 0.01

Ruberto *et al*. Supp. Fig. S5

a

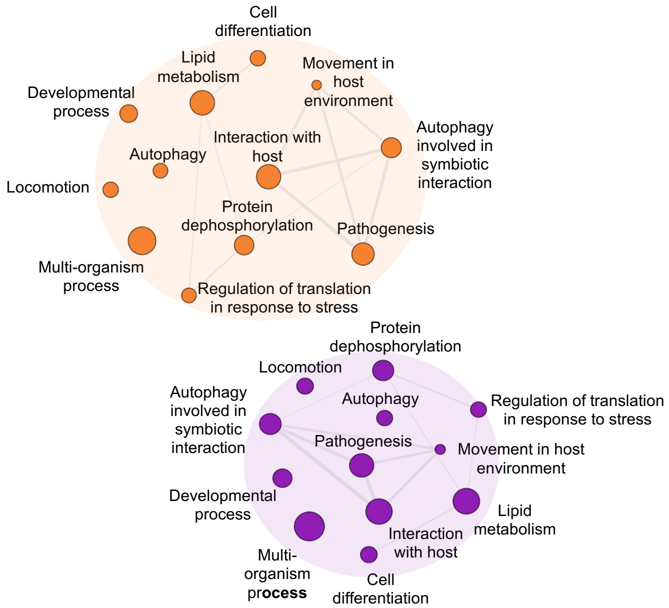

b

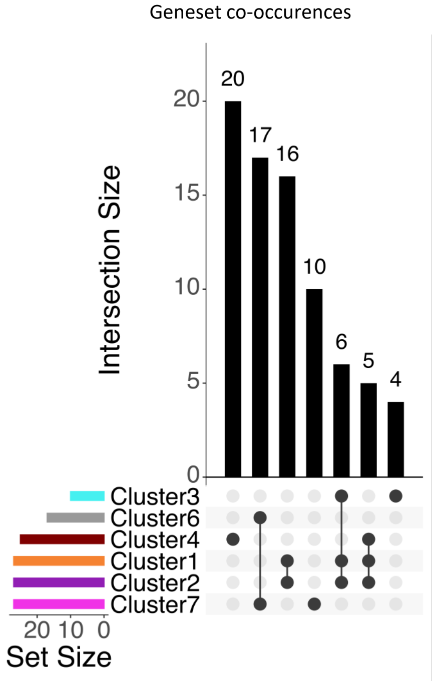

Ruberto *et al*. Supp. Fig. S6

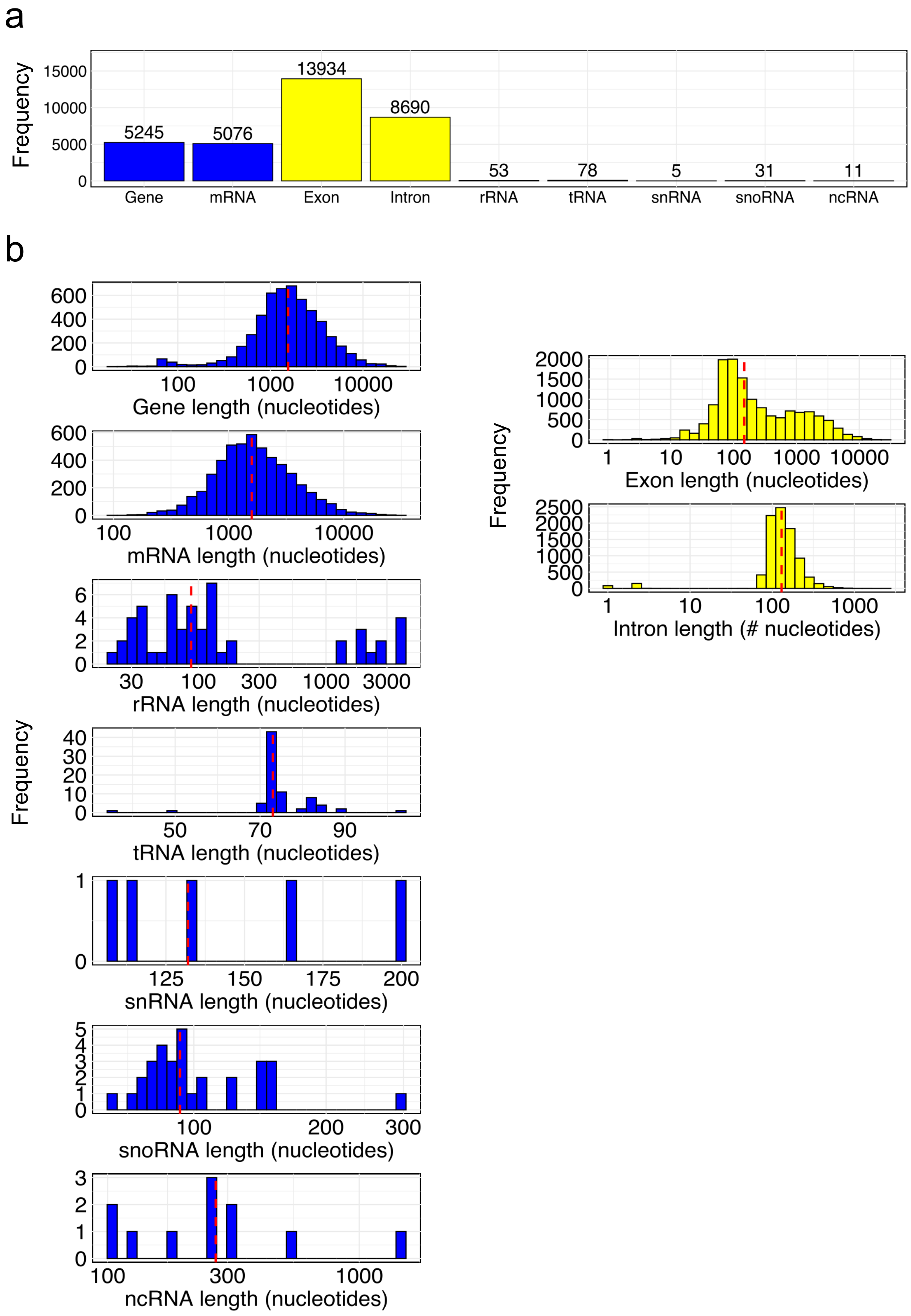

Supplement: Supplementary file 1 — Supplementary Information 1. [file 41598_2021_82914_MOESM1_ESM.pdf]
